# Temporal patterns and individual characteristics of compulsory treatment orders for mental disorders in Scotland from 2007 to 2020

**DOI:** 10.1192/bjo.2024.751

**Published:** 2024-11-11

**Authors:** Lisa Schölin, Rohan Borschmann, Arun Chopra

**Affiliations:** Centre for Cardiovascular Science, University of Edinburgh, UK; Department of Psychiatry, University of Oxford, UK; Centre for Mental Health and Community Wellbeing, University of Melbourne, Australia; Centre for Adolescent Health, Murdoch Children's Research Institute, Australia; and Justice Health Group and School of Population Health, Curtin University, Australia; Mental Welfare Commission for Scotland, UK

**Keywords:** Longitudinal data, observational study, out-patient treatment, in-patient treatment, psychiatry and law

## Abstract

**Background:**

Compulsory mental health treatment has increased globally. In Scotland, compulsory treatment for >28 days is permitted under hospital- and community-based compulsory treatment orders. Community-based compulsory treatment has not been shown to lead to improved outcomes, and scrutiny of their use is needed.

**Aims:**

To describe the trend, duration and demographic characteristics of compulsory treatment orders in Scotland over a 14-year period.

**Method:**

We conducted a retrospective analysis of order use in Scotland from 1 January 2007 to 31 December 2020, focusing on the (a) number and demographic characteristics of those treated, (b) duration, (c) extensions beyond the 6-month review point and (d) characteristics of new versus continued orders.

**Results:**

The number of individuals on a community-based order increased by 118% (571 *v.* 1243) from 2007 to 2020, compared with a 16% increase (1316 *v.* 1532) for hospital-based orders. Of orders starting in 2007, 57.3% were extended, compared with 43.7% in 2020. The median duration was 6 months for first-time orders and 9 months for subsequent orders, which were longest for males (median 11 months); those of African, Caribbean or Black (median 11 months), Asian (median 11 months) and mixed ethnicity (median 10 months); and individuals from the most deprived communities (median 10 months).

**Conclusions:**

There has been a marked rise of community-based compulsory treatment orders in Scotland. If existing trends continue, there will be more people receiving care under community-based orders than hospital-based orders, fundamentally changing the nature of involuntary treatment. Further work needs to explore associations between demographic and diagnostic characteristics on order duration.

The use of compulsory mental health treatment has increased since the mid-2000s in most jurisdictions where data are available,^1^ including Scotland.^2^ Reasons for these increases are not clear, although a study from England in 2020 indicated that changes to legal frameworks for compulsory treatment of individuals with reduced decision-making capacity, increased psychiatric morbidity in the general population and demographic changes (proportion of males, individuals from Black, Asian and minority ethnic groups, and those not born in the UK) may have contributed to increases in hospital-based detentions.^3^ International research has also emphasised that ethnic minority groups are more likely to be compulsorily treated than their White counterparts.^4^ In Scotland, data also demonstrate a clear gradient of inequality, with a higher proportion of individuals from the most deprived communities in Scotland compulsorily treated than those from less deprived areas.^2^

## Evidence around outcomes of community-based compulsory treatment

The effectiveness of community-based compulsory treatment has been the focus of considerable debate in the academic literature,^5,6^ with one commentor noting that ‘few topics in psychiatry are more controversial than compulsory community treatment [ … ] and the research into its effectiveness’ (p. 949).^7^ Previous studies have examined the impact of compulsory community treatment on outcomes of people with specific psychiatric disorders,^8^ including mortality,^9^ readmission rates^10^ and duration of in-patient stays.^8^ A 2018 systematic review and meta-analysis of 41 studies^5^ reported no consistent evidence that compulsory treatment in the community reduces either readmission rates or the length of in-patient stays. Other trials have examined the impact of compulsory community treatment on subsequent mental state, service use, social functioning and quality of life, and have also reported no demonstrable benefit of compulsory community treatment.^11^ Furthermore, a recent meta-analysis noted that predictors of outcomes vary across jurisdictions with low and high use of community treatment orders. Higher rates of use of community treatment orders was associated with more bed-days and readmissions, compared with jurisdictions where rates were low.^12^ Despite this, community treatment orders are widely accepted interventions for individuals with mental disorders, despite recent calls for them to be abolished altogether for civil sections of the Mental Health Act in England and Wales, and globally.^13,14^

## Compulsory treatment in UK legislation

In Scotland, compulsory treatment is outlined in the Mental Health (Care and Treatment) (Scotland) Act 2003 (‘the Mental Health Act’), which, at the time of writing, is under review. Under this legislation, an individual can be subjected to longer-term treatment (i.e. beyond the 28-day duration of short-term detention certificates) in hospital under a compulsory treatment order, or in community settings under a community compulsory treatment order. In England, Wales and Scotland, an initial community treatment order can last for up to 6 months. In these UK jurisdictions, community-based compulsion can be renewed, initially for a further 6 months, and thereafter on an annual basis. In contrast to other jurisdictions in the UK, compulsory treatment orders in Scotland can be hospital or community based. There is no requirement for a hospital-based order to precede a community-based order, meaning that an individual can be compulsorily treated directly through a community compulsory treatment order (see the 2003 Mental Health Act). A 2006 report noted that before the 2003 Act was enacted, estimates from the Scottish Executive suggested that approximately 200 individuals ‘at any one time’ would be subject to a community compulsory treatment order.^15^ However, the use of compulsory treatment orders and community compulsory treatment orders has increased since this time, with the most recent figures reported by the Mental Welfare Commission for Scotland (‘the Commission’) reporting that 1356 individuals were subject to a community compulsory treatment order in 2022–2023.^2^

Recent efforts to reform the Mental Health Act in England and Wales have led to suggestions that compulsory treatment in the community often lasts too long.^16^ In Scotland, the Commission has previously recommended that care plans should include revocation strategies, and the recent independent review of Scottish mental health law endorsed more monitoring and scrutiny of these instruments.^17^ It is important to acknowledge that the possible routes to compulsion in the community are different across the UK jurisdictions. In England, a patient must have been treated in hospital under Section 3 of the Mental Health Act 1983 before they can be considered for a community treatment order. In Scotland, it is possible for a person to be directly treated under compulsion in the community, which, in the most recent reporting year, was only 20 episodes out of a total 1783 compulsory treatment order episodes that year (1.1%).^2^ Little is known about the characteristics of individuals receiving treatment under compulsory treatment orders in Scotland, or the characteristics of such orders (e.g. order duration). We therefore aimed to describe the duration and characteristics of individuals who were treated under community-based and hospital-based compulsory treatment orders in Scotland between 2007 and 2020. We sought to address these specific research questions:

- What are the number and characteristics of those treated under a compulsory treatment order and how have numbers changed over time?
- What is the median duration of all compulsory treatment orders and does this vary by sociodemographic characteristics?
- What proportion of all compulsory treatment orders are extended and does this differ by sociodemographic characteristics?
- What are the number and characteristics of individuals on continued and new community compulsory treatment orders (including those varied from a hospital-based compulsory treatment order) since 2007?

## Method

### Data source

We retrospectively analysed routine data relating to compulsory treatment of individuals in Scotland over a 14-year period from 1 January 2007 to 31 December 2020. These data are routinely collected by the Commission, as compulsory treatment under a compulsory treatment order requires a formal notification to be sent to the Commission, outlining the reason for the detention in accordance with the stated criteria.

### Variables

We created five different data-sets for the analyses, corresponding to our research questions (see Supplementary Table 1 available at https://doi.org/10.1192/bjo.2024.751). We included two measures of compulsory treatment: the point prevalence of the number of individuals on compulsory treatment orders each year and annual incidence of compulsory treatment order episodes. We analysed compulsory treatment orders by (a) new (compulsory treatment orders that did not continue from the previous year) and continued orders (compulsory treatment orders which continued from the previous year), (b) first (the first compulsory treatment order for an individual during the period) and subsequent compulsory treatment orders (any compulsory treatment order that followed a first compulsory treatment order) and (c) extended compulsory treatment orders (orders that were extended beyond the initial 6-month period). Ethnicity information was obtained by matching individual records with any ethnicity information in the information management system when this was not available for the specific compulsory treatment order. Supplementary Table 1 provides proportion of records matched on ethnicity. Furthermore, in the Commission's Mental Health Act Monitoring Report 2020–2021,^2^ the Scottish Index of Multiple Deprivation (SIMD) was included for the first time, and manual cleaning of the data returned a completion rate of >90%. The SIMD uses data across seven domains (income, employment, education, health, access to services, crime and housing) of indicators that provides a relative measure of deprivation and categorises areas into zones based on deprivation ranking (into deciles or quintiles).^18^ We used the 2020 version of the SIMD rankings data zone rankings. However, the amount of missing data was too large for manual cleaning, and many older records in our data-set did not have any postcode entered, resulting in missing data (see Supplementary Table 1). For all records where a postcode was available, SIMD quintiles were matched (excluding any postcodes corresponding to a hospital or prison). Missing data for ethnicity and SIMD was excluded from subgroup analyses. Finally, although diagnosis is recorded on the detention form (using ICD-10 codes), the information system in place at the time of the study was unable to extract this information for compulsory treatment orders, and, as such, no diagnostic information was included in the analyses.

### Data analysis

We calculated descriptive statistics and compared proportions across categorical variables with chi-squared (*χ*^2^) tests. For continuous variables, the mean or median was calculated, and comparisons between groups were conducted with independent samples *t*-tests or Mann–Whitney *U*-tests. All analyses were undertaken in Microsoft Excel (version 2016 for Windows) and SPSS (version 25 for Windows). We calculated crude rates per 100 000 population, and gender- and age-specific rates with mid-year population estimates from National Records Scotland.^19^

### Ethical approval

The authors assert that all procedures contributing to this work comply with the ethical standards of the relevant national and institutional committees on human experimentation and with the Helsinki Declaration of 1975, as revised in 2013. The study draws upon routine data of individuals under compulsory treatment from a register kept by the Commission. As a result, consent for participating in the study was not obtained. This study analysed anonymised data and followed standard processes for processing personal data. The project was overseen by the Commission's Caldicott Guardian and Information Governance Manager. No ethical approval was required for the current study because the Commission has a statutory duty to collect and process these data.

## Results

### Number and demographic characteristics of compulsory treatment orders

**Fig. 1:**
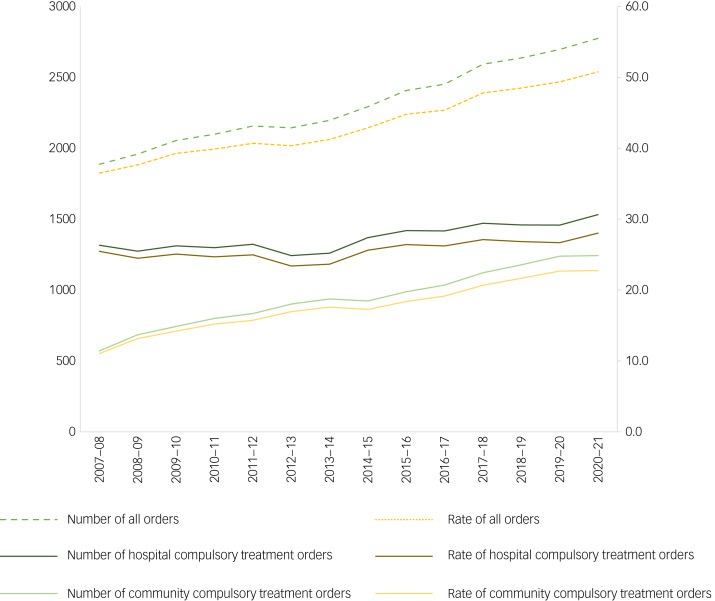
Trend in point prevalence of the number and rate per 100 000 compulsory treatment orders.

**Fig. 2:**
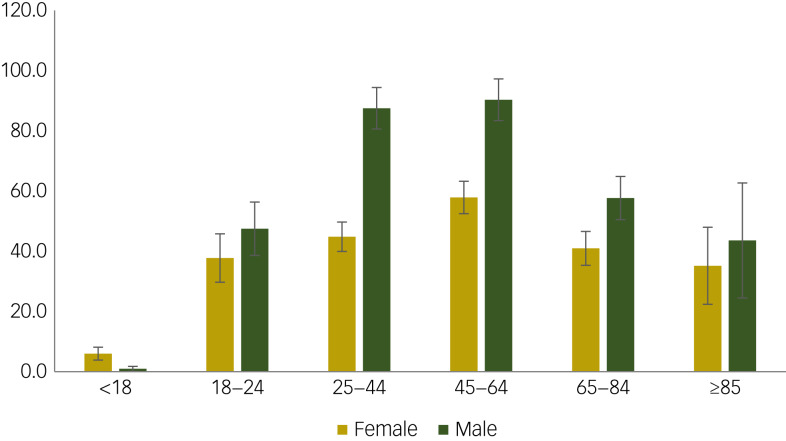
Age-standardised rates of compulsory treatment orders in 2020, by gender.

Between 2007 and 2020, the annual number of individuals on a compulsory treatment order increased by 41.7% (from 1959 to 2775), of which 44.8% were community compulsory treatment orders (Fig. 1). The number of individuals on a community compulsory treatment order increased by 118% (571 *v.* 1243), and the number of individuals on a hospital compulsory treatment order increased by 16% (1316 *v.* 1532). The overall rate of the point prevalence of compulsory treatment orders increased from 36.5 (95% CI 34.9–38.1) to 50.8 (95% CI 48.9–52.7) per 100 000, for which the rate of community compulsory treatment orders increased from 11.0 (95% CI 10.1–12.0) to 22.7 (95% CI 24.0–21.5) per 100 000 and hospital compulsory treatment orders increased from 25.5 (95% CI 24.1–26.8) to 28.0 (95% CI 29.4–26.6) per 100 000. The rate increased across all age groups for both genders over the period, with the highest rate in 2020 among males aged 25–44 years (87.5, 95% CI 80.6–94.4) and 45–64 years (90.3, 95% CI 83.4–97.3) (Fig. 2).

Compared with people receiving treatment under a hospital-based compulsory treatment order, across the entire period, a higher proportion of people receiving treatment under community-based compulsory treatment order were male (64.2% *v.* 58.0%; *P* < 0.001) and of African, Caribbean or Black (12.8% *v.* 10.9%; *P* < 0.001) and White other British (11.4% *v.* 10.3%; *P* < 0.001) ethnicity; a lower proportion were Asian (3.0% *v.* 4.5%; *P* < 0.001) and from SIMD quintile 1 (i.e. the most deprived) (39.4% *v.* 35.8%; *P* = 0.001). Individuals receiving treatment under hospital-based compulsory treatment orders were older than those on community compulsory treatment orders (47.6 *v.* 46.0 years; *P* < 0.001) (Table 1).

**Table 1:** Characteristics of compulsory treatment orders, 2007–2020

Characteristic	Grouping	Community compulsory treatment order (*n* = 13 199)	Hospital compulsory treatment order (*n* = 19 153)	All compulsory treatment orders (*N* = 32 352)	Test statistic (d.f.)	*P*-value
Gender	Female	4612 (35.8)	7674 (42.0)	12 286 (38.0)	87.2 (1)^a^	<0.0001
	Male	8587 (64.2)	11 479 (58.0)	20 066 (62.0)		
Age (years)	Mean (s.d.)	46.0 (13.8)	47.6 (18.1)	46.5 (16.5)	8.5 (32 350)^b^	<0.0001
Age group	<18 years	56 (0.4)	355 (1.9)	411 (0.3)	1074 (4)	<0.0001
	18–24 years	559 (4.2)	1551 (8.1)	2110 (6.5)		
	25–44 years	5569 (42.2)	6954 (36.3)	12 523 (38.7)		
	45–64 years	5768 (43.7)	6552 (34.2)	12 320 (38.1)		
	≥65 years	1247 (9.4)	3741 (19.5)	4988 (15.4)		
Ethnicity^c^	African, Caribbean or Black	1494 (12.8)	1762 (10.9)	3256 (11.7)	72.27 (4)^a^	<0.001
	Asian	346 (3.0)	735 (4.5)	1081 (3.9)		
	White other British	1324 (11.4)	1674 (10.3)	2998 (10.8)		
	White other	214 (1.8)	362 (2.2)	576 (2.1)		
	White Scottish	8251 (71.0)	11 681 (72.0)	19 932 (71.6)		
SIMD^d^	1 (most deprived)	864 (39.4)	1168 (35.8)	2032 (37.3)	17.8 (4)^a^	0.001
	2	603 (27.5)	943 (28.9)	1546 (28.3)		
	3	388 (17.7)	543 (16.7)	931 (17.0)		
	4	182 (8.3)	367 (11.3)	549 (10.1)		
	5 (least deprived)	156 (7.1)	240 (7.4)	396 (7.3)		

Data are displayed as *n* (%) unless otherwise indicated. SIMD, Scottish Index of Multiple Deprivation.

a. *χ*^2^.

b. *t*-statistic.

c. Excludes records that had no ethnicity recorded (13.1% of records). Note that the number of ethnicity categories differ from the analysis presented in Table 2 because they are derived from two different data-sets.

d. Excludes records where SIMD could not be matched to a valid postcode (58.7%).

### Duration of compulsory treatment orders

**Fig. 3:**
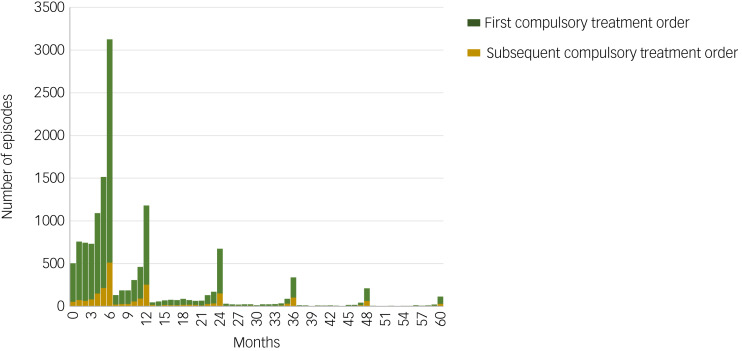
Duration of first and subsequent compulsory treatment orders. The figure excludes the smaller number of episodes lasting longer than 60 months.

Compulsory treatment orders across the study period had a median length of 6 months, with a significant difference between first-time (median 6 months, interquartile range (IQR) 4–12) and subsequent compulsory treatment orders (median 9 months, IQR = 5–23) (*P* < 0.001). Most orders (*n* = 11 661; 82.9%) were first compulsory treatment orders. Figure 3 shows that there are clear peaks in compulsory treatment orders ending at 6, 12, 24, 36, 48 and 60 months, which correspond with legally binding review points. The median length of first-time compulsory treatment orders was shorter for those aged over 65 years (median 5 months, IQR = 3–7) and longer for those of mixed ethnicity (median 11 months, IQR = 5–24). For all other demographic characteristics, the median length was consistently 6 months. Subsequent compulsory treatment orders were shorter for the youngest (<18 years) (median 5.5 months, IQR = 2–12.5) and oldest (≥65 years) (median 6 months, IQR = 4–12) age groups, and longest for 25- to 44-year-olds (median 11 months, IQR = 6–24). They were also longer for males (median 11 months, IQR = 6–24) than females (median 6 months, IQR = 5–18), and for those in SIMD quintiles 1 (median 10 months, IQR = 5–23) and 2 (median 10 months, IQR = 5–23).

### Extension of compulsory treatment orders

We explored how many compulsory treatment orders were extended beyond the 6-month review point, finding that of episodes that started in 2007, 57.3% were extended compared with 43.7% for those that started in 2020. Table 2 shows that a higher proportion of extended compulsory treatment orders than non-extended compulsory treatment orders were male (57.2% *v.* 48.1%; *P* < 0.0001); aged 18–24, 25–44 and 45–64 years (9.8% *v.* 7.7%, 36.5% *v.* 25.2 and 31.9% *v.* 24.9%, respectively; *P* < 0.0001) and in the most deprived SIMD categories (SIMD quintile 1: 35.1% *v.* 29.4%; SIMD quintile 2: 24.8% *v.* 23.0%).

**Table 2:** Demographic characteristics of extended and not-extended compulsory treatment orders, 2007–2020

Category	Grouping	Not extended (*n* = 8474)	Extended (*n* = 7843)	All compulsory treatment orders (*N* = 16 317)	*χ*^2^	*P*-value
Gender	Female	4391 (51.8)	3354 (42.8)	7745 (47.5)	133.87 (1)	<0.0001
	Male	4083 (48.1)	4489 (57.2)	8572 (52.5)		
Age, years	<18	318 (3.8)	240 (3.1)	558 (3.4)	857.62 (5)	<0.0001
	18–24	656 (7.7)	767 (9.8)	1423 (8.7)		
	25–44	2136 (25.2)	2866 (36.5)	5002 (30.7)		
	45–64	2108 (24.9)	2498 (31.9)	4606 (28.2)		
	65–84	2746 (32.4)	1359 (17.3)	4105 (25.2)		
	≥85	510 (6.0)	113 (1.4)	623 (3.8)		
Ethnicity^a^	African, Caribbean or Black	108 (1.5)	95 (1.4)	203 (1.4)	22.07 (6)	0.001
	Asian	179 (2.4)	184 (2.7)	363 (2.6)		
	Mixed	33 (0.5)	59 (0.9)	92 (0.6)		
	Other	23 (0.3)	19 (0.3)	42 (0.3)		
	White other British	617 (8.4)	495 (7.2)	1112 (7.8)		
	White other	346 (4.7)	275 (4.0)	621 (4.4)		
	White Scottish	6025 (82.2)	5750 (83.6)	11 775 (82.9)		
SIMD^b^	1 (most deprived)	1611 (29.4)	1872 (35.1)	3483 (32.2)	83.44 (4)	<0.0001
	2	1262 (23.0)	1324 (24.8)	2586 (23.9)		
	3	1024 (18.7)	954 (17.9)	1978 (18.3)		
	4	864 (15.8)	692 (13.0)	1556 (14.4)		
	5 (least deprived)	720 (13.1)	492 (9.2)	1212 (11.2)		

Data are displayed as *n* (%) unless otherwise indicated. SIMD, Scottish Index of Multiple Deprivation.

a. Excludes records that had no ethnicity recorded (28.0% of records).

b. Excludes records where SIMD could not be matched to a valid postcode (35.6%). Note that the number of ethnicity categories differ from the analysis presented in Table 1 because they are derived from two different data-sets.

### Characteristics of new versus continued community compulsory treatment orders

**Fig. 4:**
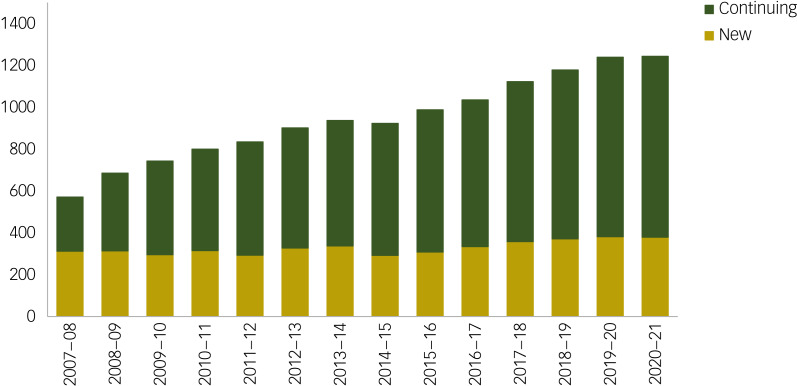
Point prevalence of individuals on a community compulsory treatment order.

The rise in community compulsory treatment orders is explained by the number of episodes that continue from the previous year. In 2007–2008, 45% of community compulsory treatment orders continued from the previous year, compared with 70% in 2020–2021 (Fig. 4). Those on continued orders were significantly older than those on new orders across all years (mean 47.1 *v.* 44.1 years; *t* = 24.06, *P* ≤ 0.0001). Significantly more individuals on continued orders were male (66.8% *v.* 60.9%; *P* < 0.001), and males were significantly younger than females, both for new orders (41.6 *v.* 47.7 years; *P* < 0.001) and continued orders (44.9 *v.* 51.6 years; *P* < 0.001). A significantly higher proportion of new orders, compared with continued orders, were among individuals of Asian (7.0% *v.* 4.7%; *P* < 0.001), White other (3.8% *v.* 2.7%; *P* < 0.001) and White Scottish (64.4% *v.* 62.9%; *P* < 0.001) ethnicity, whereas a lower proportion were African, Caribbean or Black (14.2% *v.* 10.6%; *P* < 0.001), and White other British (15.5 *v.* 14.1%; *P* < 0.001). A significantly higher proportion of new orders, compared with continued orders, were among individuals from the least deprived areas of Scotland (9.4% *v.* 6.6%; *P* = 0.033), whereas the proportion of new orders was significantly lower among those from the most deprived areas (35.8% *v.* 37.7%; *P* = 0.033).

## Discussion

To our knowledge, this is the first study to describe the characteristics of individuals treated under compulsory treatment orders in Scotland. Our study entails all records of individuals subject to compulsory treatment orders, meaning, in terms of generalisability, that it includes all individuals who experienced being on an order over the time period. Our findings indicate that, within a rising trend of compulsory treatment, there was an overrepresentation of individuals from deprived and ethnic minority communities in Scotland receiving treatment under compulsory treatment orders. Furthermore, our findings demonstrated that, although most compulsory treatment orders last no longer than 6 months, there was a trend of ending orders precisely at the statutory review points, which raises questions about whether the least restrictive practice principle is being fully implemented.

### Rising numbers of individuals on compulsory treatment orders, particularly in the community

Our analysis shows that the number of individuals treated under community compulsory treatment orders has increased significantly since 2007, and is now at similar levels to hospital-based treatment. The report from the independent review of the 1984 Mental Health Act in Scotland, which led to the current 2003 Act,^20^ noted the need for shifting resources into community-based mental health services. It is noteworthy that in the same period as we have seen community-based compulsory treatment orders rise, there has been a decline in psychiatry, addiction and intellectual disability beds in Scotland.^21^ Although causation cannot be determined, it is possible that the more limited number of beds has created a shift in placing individuals under compulsory treatment in the community rather than a hospital. If these trends continue, there needs to be increased attention to the allocation of financial resources, to ensure that community services are adequately resourced to respond to the needs of individuals with mental disorders.

With increased numbers of people treated in the community, there is also a need to explore how this is perceived by the individuals receiving such treatment. A review by Pridham et al^22^ found that although many individuals on community compulsory treatment orders felt that they were experiencing coercion, the authors highlighted findings from qualitative studies suggesting that this was contextual; i.e. despite feeling subject to coercion when on a community compulsory treatment order, this was frequently perceived as a preferable alternative to involuntary hospital admission.^22^ One of the few studies from Scotland, which included 49 individuals on community compulsory treatment orders, found that there was a sense from participants that a community order was mainly a ‘medication order’. Furthermore, participants felt that the holistic care they were anticipating from a community-based order had not materialised.^23^ As more individuals are subjected to community compulsory treatment orders, there is a need for more research on individual experiences of these orders and the care received during compulsory treatment. The increased use of community compulsion also needs to be explored, in light of findings indicating that these are not of overall benefit.^11^ Further research is needed to understand whether it is risk aversion, fear of litigation in the event of an untoward incident, a desire to ensure that patients receive robust review in increasingly under-resourced community-based services for patients with mental health conditions, or a combination of all of these, that is driving the increased use or continued use of community-based compulsion. A recent report from the Commission reports found that community-based compulsory treatment orders ensured that people received input from a community psychiatric nurse, a mental health officer (a specialist social worker) and a psychiatrist.^24^ Individuals on an order, and family members, felt that without the order they would not have access to the support they required.^24^ At the same time, there is a need to consider whether ‘revocation strategies’ (i.e. what does the person need to have demonstrated to be discharged from community compulsion) ought to be introduced into care plans, through guidance or statute.

### Measures of inequalities in compulsory care

Our findings indicate that ethnic minority communities in Scotland are experiencing compulsory treatment to a greater extent than expected based on the ethnicity data within the Scottish general population, an issue the Commission has previously highlighted.^25^ These inequalities are similar to findings in other parts of the UK^26^ and across many high-income countries.^27^

Although the database containing these data was not set up to extract information about diagnosis, and this is a limitation of the study, the need for data on diagnosis in the future is clear. Research from New Zealand^28^ has demonstrated improved outcomes in terms of reduced admissions for some individuals treated under community compulsory treatment orders with particular diagnoses, e.g. those with psychotic disorders. Further studies have shown that more targeted use of these orders is associated with more benefit.^12^This is of particular importance as targeted interventions could be considered, especially within the context of the implementation of the ICD-11. As we were unable to explore interactions between age, gender, SIMD, ethnicity and diagnosis on outcome measures, this should be included in future studies, which may require utilising other methods for coding of existing data-sets to extract diagnosis information.

This study shows, for the first time, the typical duration of compulsory treatment orders in Scotland, and highlights the pattern of ending compulsory treatment orders around the statutory review points. Most compulsory treatment orders, however, ended before or at the 6-month review point. The study demonstrates the shift toward community compulsory treatment orders and that those on community- versus hospital-based orders are, to a greater extent, male, younger, more likely to be from ethnically minority groups and more likely to be from the most deprived areas of Scotland. Compulsory treatment orders tended to last for 6 months (median length), but subsequent compulsory treatment orders tended to be longer. We can see some distinct differences in the characteristics of individuals on new and continued compulsory treatment orders, first and subsequent orders, extended and non-extended orders, and direct transfers to community-based orders. The profile of community compulsory treatment orders, overall, is different to that of those on hospital compulsory treatment orders. The current law reform process should take these findings into consideration in shaping future legislation, to ensure that individuals to the greatest extent possible have their needs and rights met when they receive involuntary treatment, which should be for the shortest time possible.

### Limitations

There are several limitations to the results of this study that need to be acknowledged. First, the data on ethnicity has been improved over time through the way we are able to link information across the Commission's data information system. However, it is not complete and may therefore not accurately reflect differences between ethnic groups, as the ethnicity of approximately 15% of the records are missing. Second, postcode information was also incomplete, contributing to a large amount of missing information for SIMD categories. Although annual data have been cleaned manually to enhance completion of postcode data,^2^ this was not feasible for over a decade's worth of records. Third, diagnostic data would enhance the findings of these results by exploring whether compulsory treatment orders for individuals with particular diagnoses are more likely to stay on an order for longer. As updates of the Commission's information system is underway and the ICD-11 is being implemented, there are opportunities to improve on the recording of this information.

### Implications

The findings from this study show that increasing numbers of individuals in Scotland are subjected to compulsory treatment orders and have implications for legislative changes through the review of the Mental Health Act in Scotland, which has been ongoing since 2019. There is a need to consider whether duties relating to compulsory treatment order review points are being met, and whether mechanisms for review need to be revised. Furthermore, our findings also suggest a need for considering revocation strategies as either guidance or as part of a care plan, and whether there is a need to place this requirement on a statutory footing and review of the length and purpose of community compulsory treatment orders, to ensure individuals are achieving real benefit.

## Supporting information

Schölin et al. supplementary material Schölin et al. supplementary material

## Data Availability

Data are not publicly available due to sensitivity reasons and ethical requirements.
