# Supplementary material for: Temporal patterns and individual characteristics of compulsory treatment orders for mental disorders in Scotland from 2007 to 2020
Source: BJPsych Open. 2024 Nov 11;10(6):e204. doi: 10.1192/bjo.2024.751 (PMC11698210; doi:10.1192/bjo.2024.751)
Supplement: Schölin et al. supplementary material [file S2056472424007518sup001.docx]

**Temporal patterns and individual characteristics of Compulsory Treatment Orders for mental disorders in Scotland from 2007 to 2020**

Lisa Schölin^1^, Rohan Borschmann^2,3,4,5,^, Arun Chopra^6^

^1^ Centre for Cardiovascular Science, University of Edinburgh, UK

^2^ Centre for Mental Health and Community Wellbeing, University of Melbourne, Australia

^3^ Department of Psychiatry, University of Oxford, UK

^4^ Centre for Adolescent Health, Murdoch Children’s Research Institute, Melbourne, Australia

^5^ Justice Health Group; School of Population Health, Curtin University, Perth, Australia

^6^ Mental Welfare Commission for Scotland, UK

**Supplementary tables**

**Table S1. Overview of datasets included in the analyses**

| **Question** | **Description** | **Start year** | **End year** | **Number** | **Ethnicity** | **SIMD** |
| --- | --- | --- | --- | --- | --- | --- |
| - How long, on average, are people subject to a Compulsory Treatment Order and does this differ by sociodemographic characteristics? - How long are first-time Compulsory Treatment Orders and are there differences in length by sociodemographic characteristics? | Compulsory Treatment Order episodes that started from 2007 onwards, excluded episodes where the episode ended because the individual died, there were errors in start and end date. | 2007^b^ | 2020 | 14,008 | 86.2% | 65.2% |
| - What proportion of all Compulsory Treatment Orders are extended and does this differ by sociodemographic characteristics? | Episodes that started before 1 January 2021 were included, as episodes after that we may not have received or there may not be an extension. Excluded episodes where death ended the episode. | 2007 | 2020 | 16,317 | 72.0% | 64.4% |
| - What is the number and characteristics of individuals on continued and new community Compulsory Treatment Orders (including those varied from a hospital-based Compulsory Treatment Order) since 2007? | Point prevalence of all individuals on a community Compulsory Treatment Order. | 2007/08 | 2020/21 | 13,199 | 83.4%^a^ | 74.6%^a^ |
| - What are the characteristics of individuals treated on a Compulsory Treatment Order in hospital and those treated in the community? | Comparison of community Compulsory Treatment Orders and hospital Compulsory Treatment Order s, using point prevalence of all individuals on a Compulsory Treatment Order. | 2007/08 | 2020/21 | 32,352 | 86.1% | 41.3% |

^a^Refers to the year 2020-21, ^b^Only episodes with a start date before 1 January 2021 were included as after that we may not have received or there might not be an extension. SIMD completion relates to the number of postcodes that simply were missing or that were a hospital postcode and therefore not relevant to calculate SIMD. Ethnicity completion excludes those where ethnicity was not reported and those that had nothing completed all together or the ethnicity form was missing and ethnicity cannot be matched with any other episodes for the same person.
